# Marsh Plant—Relation to Malarial Diseases

**Published:** 1874-01

**Authors:** 


Chicago Society of Physicians and Surgeons.—Dr. John Bartlett read a paper entitled, "On a Marsh Plant from the Mississippi River Ague Bottoms, with a Consideration of its Genetic Relation to Malarial Diseases," the growth of which he discovered to be simultaneous with the appearance of malarial fevers, containing cells of exceeding minuteness, which possessed the power of rapid multiplication. These had been detected by Dr. Bartlett, in his own blood, and in that of several ague patients, and had been seen by him multiplying with great rapidity, and producing products similar to those recognized as peculiar features of the plant, in the blood, the urine and the saliva. On the conclusion of the reading, the members of the Society, and invited guests, inspected the microscopic preparations, colored drawings of the plant, and specimens of the plants themselves, growing in their native soil, which were intended to illustrate the reading.—*Chicago Medical Journal.*

Persimmon Coffee.—Persimmon coffee is much preferred to the burned bean variety in Georgia. A gentleman of that State has taken out a patent for making it, and describes his process as follows : "My mode of preparation consists of steaming the fruit for half an hour in a boiler, and after crushing them I throw them into a tank of water, and the seed are easily washed out, as their own specific gravity carries them to the bottom, and the pulp can be floated off. The seed should then be spread out in the sun to dry for three or four weeks, and then parched and ground similar to any other coffee, care being taken to have them parched sufficiently to grind easily. The seed by this process can be obtained, where the fruit is plenty, at a cost of two cents per pound, and, if properly prepared, are equal in all respects to good Java coffee."—*Ibid.*

